# Reproduction of Bones and Joints after Their Removal in Cases of Whitlow

**Published:** 1858-02

**Authors:** 


*Reproduction of Bones and Joints after their removal in cases of Whitlow.*—We notice in the January number of the Peninsular Journal, a number of cases quoted from the California Medical Journal and Review, in which entire phalanges, which have been removed for disease or injury, have been reproduced, even to their articular surfaces, forming a joint as perfect as before. The author, Dr. H. H. Toland, of San Francisco, does not appear to be aware of communications which have appeared on this subject in this Journal, by Prof. Hamilton, and in the American Journal, by Prof. Dudley, of Lexington, Ky., but lays claim to the discovery of this reproduction of joints; we quote the following from Dr. Toland's article:

As the bone is formed by the periosteum, if the latter be not removed, and the soft parts are kept extended, the bones as well as the joints are reproduced, and the part restored to its former usefulness. It has long been known that bone will be reproduced provided the periosteum remains, and we only claim to have made the discovery that the joints are also, by proper management, restored, even under the most unfavorable circumstances. Whether they are as perfect as the original, possessing cartilages, ligaments, and synovial fluid, we are unable to say; but we know that they possess motion, and sufficient strength to perform the function of the most perfect joints.

Dr. Dudley mentioned his practice in such cases, in the Transylvania Med. Journal, for Dec., 1849, but published no illustrative cases. Prof. Hamilton, however, in May, 1850, published a case analagous to those reported by Dr. Toland, in this Journal, which we extract:

Catherine Dolan, aged 24, was admitted into the hospital, Dec. 25, 1849. Thumb. The bone was necrosed, and on the 29th, I extracted the phalanx entire. The inflammation having considerably subsided by the third of January, five days after the operation, I applied a tape roller the whole length of the thumb, and moderately tight. This was continued with occasional intermissions, for two months, when a new phalanx was found to have been formed, of the same length, breadth and form as the original phalanx; the articulating surface was also reformed, and the flexor and extensor tendons so attached as that the motions of the joint were perfect.

This result is not now for the first time discovered. More than a year since, Prof. Dudley, of Lexington, informed me in a private communication, that he had been able to reconstruct a phalanx, when the bone had been entirely removed, by the continued application of a roller. To Dr. Dudley, therefore, is the profession indebted for this interesting pathological discovery.

Thus our readers will see that though an important and interesting observation, we are compelled to give the credit of the discovery to Dr. Dudley, and we believe that Dr. Hamilton was the first to publicly confirm his statement, and the very first who published a case. We are somewhat surprised that Dr. Toland did not know of this discovery by Dr. Dudley, as in addition to the articles in the Transylvania and the Buffalo Journals, an article appeared at a later date, in the American Journal of Medical Sciences, of the existence of which Dr. Toland and the editors of the Peninsular Journal seem to be equally unaware. The treatment employed by Dr. Toland is essentially the same as that recommended by Prof. Dudley, and followed with such good success by Prof. Hamilton, the only difference being that the former uses pasteboard splints, while the latter uses a narrow roller, the object of each method being merely to support the soft parts. F.

